# Neurological Disorders in Central Spain, Second Survey: Feasibility Pilot Observational Study

**DOI:** 10.2196/10941

**Published:** 2019-01-10

**Authors:** Jesús Hernández-Gallego, Sara Llamas-Velasco, Felix Bermejo-Pareja, Saturio Vega, Ester Tapias-Merino, Emiliano Rodríguez-Sánchez, Elina Boycheva, José Ignacio Serrano, Juan-Francisco Gil-García, Rocio Trincado, José-María Vizcaino Sánchez-Rodrigo, Jesús Cacho, Israel Contador, Sara Garcia-Ptacek, Fernando Sierra-Hidalgo, Esther Cubo, Eva Carro, Alberto Villarejo-Galende, Rosalía García García-Patino, Julián Benito-León

**Affiliations:** 1 Department of Neurology University Hospital “12 de Octubre” Madrid Spain; 2 Spanish Network for Biomedical Research in Neurodegenerative Diseases Carlos III Research Institute Madrid Spain; 3 Department of Medicine Faculty of Medicine Complutense University of Madrid Madrid Spain; 4 Group of Neurodegenerative Diseases Research Institute University Hospital “12 de Octubre” Madrid Spain; 5 Arevalo Health Center Arevalo (Ávila) Spain; 6 Comillas Health Center Madrid Spain; 7 La Alamedilla Health Center La Alamedilla (Salamanca) Spain; 8 Neural and Cognitive Engineering Group Centro de Automática y Robótica Spanish National Research Council Arganda del Rey (Madrid) Spain; 9 Cantalejo Health Center Cantalejo (Segovia) Spain; 10 Fuentelarreina Health Center Madrid Spain; 11 Department of Neurology University Hospital of Salamanca Salamanca Spain; 12 Department of Basic Psychology, Psychobiology and Methodology of Behavioural Sciences University of Salamanca Salamanca Spain; 13 Department of Geriatric Medicine Karolinska University Hospital Huddinge Sweden; 14 Division of Clinical Geriatrics Department of Neurobiology, Care Sciences and Society Karolinska Institutet Stockholm Sweden; 15 Department of Neurology University Hospital Infanta Leonor Madrid Spain; 16 Department of Neurology University Hospital “General Yagüe” Burgos Spain

**Keywords:** dementia, essential tremor, headache, longitudinal study, mild cognitive impairment, NEDICES, observational study, Parkinson’s disease, pilot study, population-based study, stroke

## Abstract

**Background:**

The Neurological Disorders in Central Spain, second survey (NEDICES-2) is a population-based, closed-cohort study that will include over 8000 subjects aged ≥55 years. It will also include a biobank.

**Objective:**

The objective of this study was to evaluate all major aspects of the NEDICES-2 (methods, database, screening instruments, and questionnaires, as well as interexpert rating of the neurological diagnoses) in each one of the planned areas (all of them in central Spain) and to test the possibility of obtaining biological samples from each participant.

**Methods:**

A selection of patients and participants of the planned NEDICES-2 underwent face-to-face interviews including a comprehensive questionnaire on demographics, current medications, medical conditions, and lifestyle habits. Biological samples (blood, saliva, urine, and hair) were also obtained. Furthermore, every participant was examined by a neurologist.

**Results:**

In this pilot study, 567 study participants were enrolled (196 from hospitals and 371 from primary care physician lists). Of these 567, 310 completed all study procedures (questionnaires and the neurological evaluation). The study was time-consuming for several primary care physicians. Hence, a few primary care physicians from some areas refused to participate, which led to a reconfiguration of study areas. In addition, the central biobank needed to be supplemented by the biobanks of local Spanish National Health System hospitals.

**Conclusions:**

Population-based epidemiological surveys, such as the NEDICES-2, require a pilot study to evaluate the feasibility of all aspects of a future field study (population selection, methods and instruments to be used, neurological diagnosis agreement, and data collection).

## Introduction

A pilot study is usually recommended before undertaking epidemiological research in large populations to study neurological, psychiatric, or aging-related diseases [1-9]. Surveys investigating neurological diseases are especially difficult because of the need for an expert diagnosis, as a sizable proportion of neurological disorders do not have diagnostic biomarkers [10]. The difficulty increases when epidemiological surveys require upfront screening to obviate the workload of a 2-phase survey [2]. In this type of neurological research, pilot studies prior to the field survey become mandatory.

The original *Neurological Disorders in Central Spain,* first survey (NEDICES-1) was a closed population-based study, which followed a cohort over 13 years [2,3,11,12]. The NEDICES has produced high-quality epidemiological research on different neurological disorders with >70 peer-reviewed publications regarding stroke, dementia, parkinsonism, tremor, and various aspects of aging and mortality. The main limitation of the NEDICES-1 was the few laboratory data we obtained from participants; to overcome this limitation, we have established a new observational survey, the *Neurological Disorders in Central Spain*, second survey (NEDICES-2).

The main differences between the NEDICES-1 and NEDICES-2 are the following. First, in the NEDICES-2, we selected participants through primary care physicians, instead of using the census as in the NEDICES-1. Currently, the Spanish National Health System includes virtually all Spanish legal residents and immigrants. We used computerized data of citizens assigned to primary care physicians because they perform their clinical work at Spanish National Health System centers. Second, this new cohort of participants comprises subjects aged >54 years. The younger cohort was not included in the original survey. Third, a tissue bank (biobank) of participants was created. Fourth, the study areas were not the same as those of the previous NEDICES survey, although similarly located in central Spain. Finally, in the NEDICES-2, the computerized registry of clinical and biological data is centralized in a specific website.

The main objective of our pilot study was to evaluate all major aspects of the NEDICES-2 survey (methods, database, screening instruments, and questionnaires, as well as interexpert rating of neurological diagnoses) and to test the possibility of obtaining biological samples from each participant. Another important objective of this pilot study was to assess the levels of cooperation among potential participants and identify and resolve newly arising problems.

## Methods

### Design

The coordinating center (Research Institute of the University Hospital “12 Octubre” in Madrid) of the NEDICES-2 designed all aspects of the survey during 2011-2012, advised by participating primary care physicians. The methods and protocols were analogous to the NEDICES-1 survey [2,3,11,12].

### Objectives

The NEDICES-2 survey aims to establish a population-based cohort to investigate major age-related neurological disorders (essential tremor, Parkinsonism, stroke, mild cognitive impairment [MCI], and dementia), including the risk factors and possible biomarkers for such neurological diseases. In addition, we aim to confirm the general findings of the NEDICES-1 survey with a new larger cohort, including persons in late adulthood (age 55-64 years) and to assess the possible changes in the neurological diseases incidence over time. Finally, one important aim of this study is to obtain biological samples (blood, urine, hair, and saliva).

### Population and Study Areas (Pilot Study Selection)

The NEDICES-2 survey aims for a baseline cohort population of approximately 8000 participants; this number was calculated to adequately detect Parkinson disease, which has the lowest prevalence and incidence of all neurological disorders studied in this research [13,14]. We expect a similar attrition as happened in the NEDICES-1 survey [12]. The areas were selected to represent rural, semirural, and urban populations.

The composition by age reflects the general Spanish population aged >54 years (30% in the strata of 55-64 years); this age composition is like other European cohorts such as the Rotterdam study [15]. The NEDICES-2 population will be selected from primary care physicians’ lists to obtain a random group (400-600 subjects per primary care physician’s list) representative for age (5-year strata) and sex of subjects aged 55-84 years and of all subjects aged >84 years.

The coordinating center of the NEDICES-2 set up the pilot field study and selected the study protocol (questionnaires, scales, and examinations) with few differences from the NEDICES-1 survey. Furthermore, it developed the telematic utilities for the survey—specific email, Skype conferences, information website, and an electronic platform—to collect study data with privacy requirements. The coordinating center also conducted the pilot study.

The coordinating center selected 7 areas to survey participants for the pilot study: Fuentelarreina and Comillas (central Madrid), Las Margaritas (Getafe, peripheral Madrid), Arganda del Rey (suburban Madrid, semirural area), Cantalejo (Segovia, rural area), Burgos county (rural area), Arévalo (Ávila, semirural area), and Pizarrales (Salamanca, urban area). The participants were randomly selected (choosing 5 of 20) and stratified by sex and age (in 5-year age spans) to be evaluated by 7 primary care physicians. Participants were considered eligible if they had lived in rural or urban areas for >6 months and did not anticipate a serious illness that could cause death within the next year. In addition, for this pilot study, we recruited patients from the outpatient neurology clinics of both the University Hospital “12 de Octubre” in Madrid and the Burgos University Hospital in Burgos. These comprised patients diagnosed with the following neurological disorders: essential tremor, Parkinsonism, stroke, headache, MCI, and dementia.

### Questionnaires and Screening Methods

We administered 3 different types of questionnaires. First, lay interviewers (mostly students, not in medicine) administered general questionnaires, including information on the demographic and social aspects of participants. In addition, these questionnaires included screening questions (see below) or brief neuropsychological batteries for detecting or confirming neurological diseases, such as essential tremor [16-18], Parkinsonism [13,14,19], stroke [20,21], or MCI or dementia (37-item version of the Mini-Mental State Examination, clock drawing test, 11-item version of the Pfeffer Functional Activities Questionnaire, Word Accentuation test, verbal fluency [animals and fruits], Trail-Making test, delayed late memory tests, logical memory, and nomenclature and images) [22-27], which had been used in the NEDICES-1. Second, primary care physicians also administered a questionnaire with anthropometric data, general health status, cardiovascular risk factors, previous illnesses, clinical comorbidity [28], and current medications. Furthermore, primary care physicians took standard biochemical specimens (blood and urine) for registration and for the biobank (in some cases, hair and saliva were also included). Finally, a self-reported questionnaire was also completed by each study participant, providing personal data, professional background, education and studies, measurements of subjective health (global, age-comparative, and time-comparative self-rated health) [29], generic health-related quality of life (European Quality of Life Scale) [30], Epworth sleepiness scale [31], physical activity [32], headaches [33], the Beck Depression Inventory scale [34], social relationships of participants, and information about their lifestyle (drugs, tobacco, coffee, and alcohol consumption).

Screening methods for the main neurological disorders were like those used in the NEDICES-1 survey [2,3,11,12]. A random sample of approximately 4% of those who had screened negative in the NEDICES-1 was selected and contacted to assess the performance of these screening methods [1]. Of 205 subjects who were contacted, 183 were successfully scheduled for a neurological examination by a senior neurologist [1]; they all were examined, and none was found to have essential tremor, parkinsonism, or stroke; however, 1.1% (2/183) of subjects were found to have “mild dementia” (95% CI 0.3%-3.9%) [1].

In the NEDICES-1, we included a question for essential tremor (“have you ever suffered from tremor of the head, hands, or legs that has lasted longer than several days?”) [17,18]. In addition, 3 questions were administered to screen for parkinsonism (questions about the previous diagnosis of Parkinson disease, tremor, and bradykinesia) [13,14]. Furthermore, we used a validated 9-item questionnaire aimed at identifying parkinsonism-related symptoms [19]. The screening instrument for stroke was a Spanish adaptation of the questionnaire used for screening in the World Health Organization Multinational Monitoring of Trends and Determinants in Cardiovascular disease project [35]. The main screening instruments for MCI or dementia included the Spanish adaptation of a cognitive test (a 37-item version of the Mini-Mental State Examination) and an instrumental activity of daily living scale (11-item version of the Pfeffer Functional Activities Questionnaire) [22,24]. The sensitivities of both the 37-item version of the Mini-Mental State Examination and the Pfeffer Functional Activities Questionnaire Scale are >90% [36].

### Ethical Aspects

All participants included in the study gave their written informed consent after full explanation of the procedure. The study, which was conducted in accordance with the principles of the 1975 Declaration of Helsinki, was approved by the ethical standards committee on human experimentation at the University Hospital “12 de Octubre” (Madrid). Written (signed) informed consent was obtained from all enrollees. The data collection and biobank procedures conformed to the Spanish law.

### Statistical Analyses

Statistical analyses were performed using SPSS Version 21.0 (IBM Corp). All tests were 2-sided, and the significance was accepted at the 5% level (alpha=.05). Continuous variables were compared using Mann-Whitney *U* test because they were all nonnormally distributed. Furthermore, chi-square test was used to analyze categorical variables.

## Results

### Population

**Figure 1:**
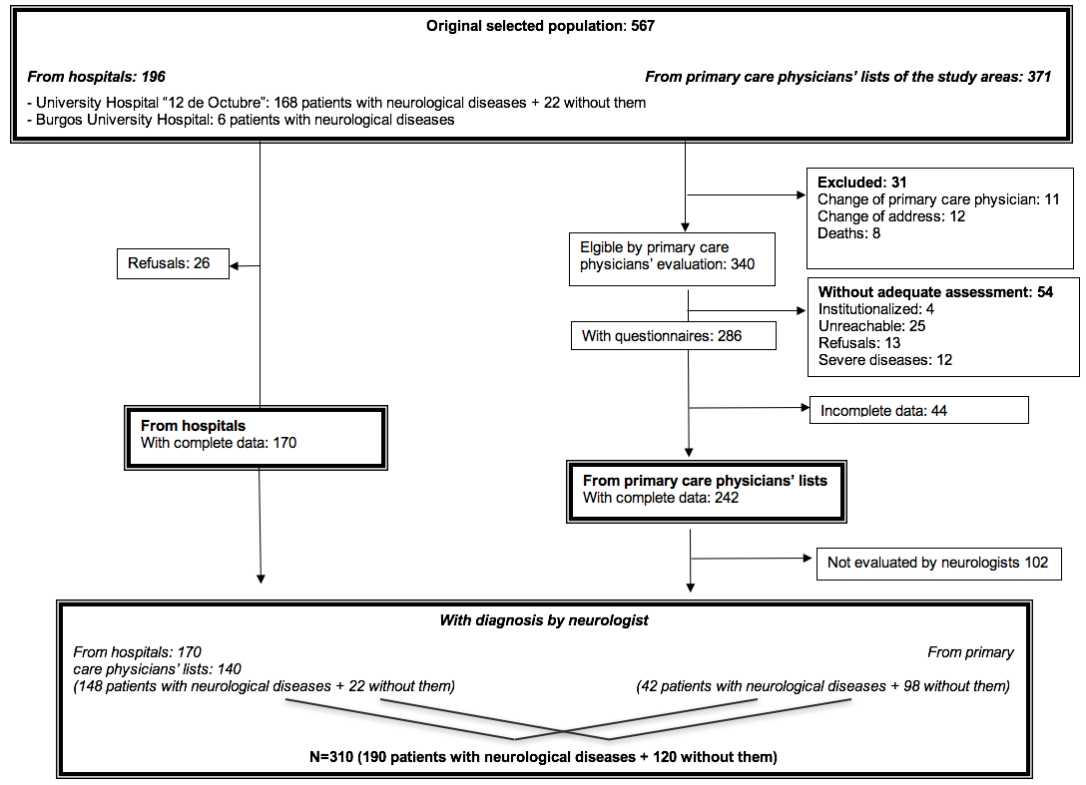
Flow chart of the Neurological Disorders in Central Spain, second survey (NEDICES-2), feasibility pilot study.

Figure 1 shows the flowchart of the study. The originally selected population for the pilot study was of 567 participants. Of them, 34.6% (196/567) were recruited from the outpatient neurology clinics of both the University Hospital “12 de Octubre” in Madrid, which provided 168 patients with neurological diseases and 22 patients without them after a careful examination, and the Burgos University Hospital in Burgos, which provided 6 patients with neurological diseases. The remaining 65.4% (371/567) participants were selected from primary care physicians’ lists of the study areas.

Of the subjects recruited from hospitals, 13.3% (26/196) refused to participate; meanwhile, 86.7% (170/196) were adequately evaluated (complete data and assessment by a neurologist). Of the 371 participants selected from primary care physicians’ lists, 8.3% (31/371) were excluded because of change of primary care physician, change of address, and deaths. Of the remaining 340 who were eligible by the primary care physician assessment, 15.9% (54/340) were excluded because of inadequate evaluation (institutionalization, unreachable, refusals, and severe diseases). The remaining 286 fulfilled or were administered the questionnaires, but 15.4% (44/286) were excluded because of incomplete data. Finally, 242 participants had complete data; however, only 57.9% (140/242) participants were eligible for the final analyses because neurologists could not evaluate 42.1% (102/242) for several reasons.

Thus, the final sample consisted of 310 participants (61.3%, 190/310, with neurological diseases and 38.7%, 120/310, without them) who presented all the required data (3 questionnaires, neurological evaluation, and biobank donation; Table 1). A higher proportion of participants with neurological diseases were more likely to have depression or depressive symptoms, cataracts, and score worse on screening tests for cognitive disorders (37-item version of the Mini-Mental State Examination and 11-item version of the Pfeffer Functional Activities Questionnaire). In addition, as expected, they scored higher in the Parkinson disease screening test and rated their health as bad or very bad. On the other hand, they were more likely to be more sedentary. Table 2 shows the final sample of participant distribution according to neurologists’ diagnosis.

**Table 1:** Baseline demographics and a selection of clinical characteristics of the final sample of participants (N=310).

Characteristics			Without neurological diseases (n=120^a^)	With neurological diseases (n=190^b,c^)	*P* value^d^	
Age (years), mean (SD); median			70.7 (9.4); 69	71.7 (9.4); 73	.28	
**Sex, n (%)**						.92
	Men		55 (45.8)	86 (45.3)		
	Women		65 (54.2)	104 (54.7)		
Years of education in years^e^, mean (SD); median			9.9 (5.6); 8.0	8.4 (5.2); 8.0	.52	
**Main nonneurological disorders^e^, n (%)**						
	Diabetes		21 (18.4)	38 (20.7)	.64	
	Arterial hypertension		62 (53.9)	99 (53.5)	.95	
	Hypercholesterolemia		56 (48.3)	87 (47.0)	.83	
	Heart diseases		20 (20.4)	34 (22.2)	.73	
	Osteoarthritis		59 (51.8)	95 (52.5)	.90	
	Cancer		18 (16.1)	22 (12.2)	.35	
	Cataracts		30 (26.3)	74 (41.8)	.007	
	Chronic pulmonary disease		16 (14.3)	25 (13.7)	.90	
	Depression		17 (14.9)	50 (27.6)	.011	
	Deafness		17 (15.2)	57 (31.5)	.002	
**Lifestyle variables^e^**						
	Sleeping hours, mean (SD); median		7.0 (1.4); 7.0	7.2 (1.6); 7.0	.47	
	Ever smoker (ex-smoker plus current smoker), n (%)		1 (11.3)	11.0 (8.9)	.12	
	Ever drinker (ex-drinker plus current drinker), n (%)		67 (57.3)	103 (54.8)	.67	
	**Physical activity, n (%)**					.004
		Inactive	60 (52.2)	130 (69.1)		
		Moderately inactive	6 (5.2)	15 (8.0)		
		Moderately active	22 (19.1)	20 (10.6)		
		Active	27 (23.5)	23 (12.2)		
**Self-rated health^e^, n (%)**						.01
	Very good		15 (12.8)	8 (4.2)		
	Good		55 (47.0)	72 (38.1)		
	Fair		41 (35.0)	94 (49.7)		
	Poor		5 (4.3)	13 (6.9)		
	Very poor		1 (0.9)	2 (1.1)		
**Screening tests for cognitive disorders^e^, mean (SD); median**						
	37-item version of the Mini-Mental State Examination		32.2 (4.8); 33.0	30.7 (5.7); 32.0	.02	
	11-item version of the Pfeffer Functional Activities Questionnaire		1.1 (4.0); 0	3.2 (6.0); 0	<.001	
Parkinson disease screening test, mean (SD); median			1.0 (1.2); 1.0	2.8 (2.4); 2.0	<.001	
Headache (yes vs no), n (%)			29 (27.4)	57 (34.3)	.26	
Beck Depression Inventory scale, mean (SD); median			7.1 (5.6); 6.0	9.7 (6.3); 9.0	<.001	

^a^98 from hospitals + 22 from primary care physicians’ lists.

^b^148 from hospitals + 42 from primary care physicians’ lists.

^c^15 patients had more than one disorder.

^d^Continuous variables were compared using the Mann-Whitney *U* test because they were all nonnormally distributed. Furthermore, the chi-square test was used to analyze categorical variables.

^e^Data on some participants were missing.

**Table 2:** Final participant sample (N=310) distribution according to neurologists’ diagnosis.

Characteristics		Without neurological diseases (n=120)			With neurological diseases^a^ (n=190)											
					Headache (n=37)		Dementia (n=28)		Parkinson disease (n=35)		Stroke (n=43)		Mild cognitive impairment (n=23)			Essential tremor (n=39)
Age (years), mean (SD)			70.1 (9.6)	66.4 (10.5)		78.2 (6.8)		71.5 (10.2)		71.1 (8.4)		75.6 (6.5)			71.4 (8.1)	
**Gender, n (%)**																
	Men	55 (45.8)			14 (37.8)		11 (39.3)		21 (60.0)		23 (53.5)			12 (52.2)		14 (35.9)
	Women	65 (54.2)			23 (62.2)		17 (60.7)		14 (40.0)		20 (46.5)			11 (47.8)		25 (64.1)

^a^Fifteen participants had more than one disorder.

All selected areas participated in the survey (participant evaluation and acquisition of biological samples), but the quality of the information obtained and the clinical workload for primary care physicians was quite varied, as was the biobank established in each local area. Primary care physicians of Arganda del Rey, Las Margaritas (Madrid), and Pizarrales (Salamanca) had difficulties carrying out the survey because of high clinical load, and therefore, they refused to participate in this pilot study; these areas were then replaced by La Alamedilla (Salamanca, urban area), Calesas, (urban area, Madrid), Valladolid (urban area), and Cantalejo (rural area, Segovia).

### Development of the Pilot Study

Each primary care physician invited selected subjects from among his or her patient list to join in the survey through a phone call or a letter. The duration of the interviews with participants was variable (10-20 minutes). Most participants signed the informed consent for both clinical participation and donation to biobanks. The pilot study showed that the central biobank faced practical difficulties such as shortage of staff, high costs, and difficulties with sample arrangements in the University Hospital “12 de Octubre” biobank. The coordinating center overcame this problem by changing the initial survey design to use local biobanks as supplements to the central biobank (except for Madrid, Ávila, and Segovia).

The training of interviewers was satisfactory. Interviewer questionnaires were digitized and sent to the central website. Most of the participants’ self-report questionnaires had to be completed on paper and sent to the coordinating center in this format. Evaluations by lay interviewers lasted approximately 1 hour and 15 minutes, sometimes up to 3 hours, with a break. Participants with possible neurological disorders received a second evaluation performed by a neurologist.

The comprehension of questionnaires was generally adequate, with some exceptions. The Beck Depression Inventory scale [34] was difficult to understand for many participants, and the coordinating center replaced it by the Center for Epidemiologic Studies Depression Scale [37] for the future field study. The 37-item version of the Mini-Mental State Examination, 11-item version of the Pfeffer Functional Activities Questionnaire, Word Accentuation test, verbal fluency, Trail-Making test, delayed late memory tests, logical memory, and nomenclature and images [22-25] allowed us to establish the psychometric cuts for screening for dementia, obtaining sensitivity >95% with high specificity.

Once the pilot study was completed, it was decided to compile each subject’s evaluations; these summary sheets were sent to each primary care physician for them to discuss with each subject, explain the results obtained, and thank them for their collaboration.

### Interrater Agreement in the Clinical Diagnosis of Neurologists Who Will Participate in the Field Study

The interrater agreement of cognitive status and tremor disorders have been published elsewhere [16,38]. Briefly, to assess the diagnostic agreement of cognitive status (dementia, MCI, and normal cognition) among neurologists, medical histories of 30 individuals were provided to 27 neurologists (19 consultant neurologists and 8 neurology residents) [38]. Overall, the interrater agreement on cognitive status was κ=.76 (95% CI 0.65-0.86), being slightly higher among junior neurologists (κ=.85, 95% CI 0.73-0.95) than among seniors (κ=.71, 95% CI 0.59-0.83) and residents (κ=.69, 95% CI 0.54-0.81), but without statistical significance among groups [38]. Dementia severity showed an overall kappa of .34, .44, and .64 for mild, moderate, and severe dementia, respectively [38]. Furthermore, clinical histories and standardized videotaped neurological examinations of 26 individuals (11 with essential tremor, 7 with Parkinson disease, 3 diagnostically unclear, 4 normal, and 1 with a tremor disorder other than essential tremor) were provided to 7 consultant neurologists, 6 neurology residents, and 5 neurology research fellows. For each of the 26 individuals, neurologists were asked to assign a diagnosis of “essential tremor” or “no essential tremor” using the diagnostic criteria proposed by the Movement Disorders Society [39]. The overall kappa was .61 (substantial agreement), with no differences among consultant neurologists (κ=.60), neurology residents (κ=.61), and neurology research fellows (κ=.66) in subgroup analyses [16].

## Discussion

A pilot study is the first step in many types of epidemiological studies such as cross-sectional and longitudinal surveys [2-5,9], case-control studies [40], and research investigations [41]. It is unwise to establish a complex survey without an adequate pilot study, as was demonstrated by this study.

Participants, in general, were more cooperative than expected. The main difficulties in the study were unexpected. Specifically, the study was time consuming for primary care physicians, as they had to explain it to participants, obtain informed consents, and complete a summary of their medical history. However, during the design of the NEDICES-2 survey, the coordinating center had erroneously considered that the 3 tasks could be performed in 10 minutes because the lay interviewer had given written information to participants and because there was a specific website with an explanation of the study.

Primary care physicians have a heavy workload in Spain. Many participants required a lengthy explanation of the survey, despite the good explanation on the website. With the public funds that we obtained, we could pay external collaborators as interviewers, but the Spanish National Health System research policy does not permit payment to primary care physicians, even if they devote extra time beyond their working hours. All this caused 3 primary care physicians’ teams to leave this study. Thus, the coordinating center had to replace these primary care physicians’ study areas by others, even in other geographical areas, to overcome this problem.

Another important difficulty was obtaining biological samples to establish the central biobank at the University Hospital “12 de Octubre” in Madrid. The pilot study showed that the only possible way to address this problem was to send all biological samples to each local Spanish National Health System hospital laboratory (for registration and to establish a local biobank). Otherwise, the pilot study was successful, partly because the methods were analogous to the NEDICES-1. Only a few changes in the screening questionnaires and scales were made after the pilot survey. Moreover, several tests were established for the detection of MCI and dementia.

Substantial agreement was demonstrated for the diagnosis of cognitive status (dementia, MCI, and normal cognition) among neurologists of different levels of experience [38]. The agreement rate was lower in the diagnosis of dementia severity. With respect to tremor disorders, substantial agreement was also demonstrated for the diagnosis of essential tremor among neurologists of different levels of expertise [16]. However, the agreement was lower than that previously reported using the Washington Heights–Inwood Genetic Study of Essential Tremor criteria [42].

In conclusion, we feel that it is impossible to undertake a large and complex neuroepidemiology survey, such as the NEDICES-2, without a pilot study. It is mandatory first to test the feasibility of all aspects of a future field study—population selection, methods, instruments to be used, neurological diagnosis agreement, and data collection. This pilot study reveals some serious deficiencies in the selected areas (the ability of overburdened primary care physicians to collaborate) and the biological bank configuration that could be solved by the coordinating center.

## Abbreviations

MCI: mild cognitive impairment
NEDICES-1: The Neurological Disorders in Central Spain, first survey
NEDICES-2: The Neurological Disorders in Central Spain, second survey
